# Natural Choline from Egg Yolk Phospholipids Is More Efficiently Absorbed Compared with Choline Bitartrate; Outcomes of A Randomized Trial in Healthy Adults

**DOI:** 10.3390/nu11112758

**Published:** 2019-11-13

**Authors:** Lotte Smolders, Nicole J.W. de Wit, Michiel G.J. Balvers, Rima Obeid, Marc M.M. Vissers, Diederik Esser

**Affiliations:** 1 AAK, Department of Special Nutrition, AAK Netherlands BV, Zaandijk, Kreeftstraat 1, 1544 CK Zaandijk, The Netherlands; Marc.vissers@aak.com; 2 Wageningen Food and Biobased Research, Wageningen University & Research, 6708 WG Wageningen, The NetherlandsDiederik.esser@wur.nl (D.E.); 3 Division of Human Nutrition and Health, Wageningen University and Research, 6708 WE Wageningen, The Netherlands; Michiel.balvers@wur.nl; 4 Department of Clinical Chemistry and Laboratory Medicine, Saarland University Hospital, D-66421 Homburg, Germany; Rima.Obeid@uks.eu

**Keywords:** choline, natural, phospholipids, DHA, absorption

## Abstract

Choline is a vitamin-like essential nutrient, important throughout one’s lifespan. Therefore, choline salts are added to infant formula, supplements and functional foods. However, if choline is present in a natural form, e.g. bound to phospholipids, it may be more efficiently absorbed. The study’s aim was to evaluate if choline uptake is improved after consumption of an egg yolk phospholipid drink, containing 3 g of phospholipid bound choline, compared to a control drink with 3 g of choline bitartrate. We performed a randomized, double blind, cross-over trial with 18 participants. Plasma choline, betaine and dimethylglycine concentrations were determined before and up to six hours after consumption of the drinks. The plasma choline response, as determined by the incremental area under the curve, was four times higher after consumption of the egg yolk phospholipid drink compared with the control drink (*p <* 0.01). Similar outcomes were also observed for choline’s main metabolites, betaine (*p <* 0.01) and dimethylglycine (*p =* 0.01). Consumption of natural choline from egg yolk phospholipids improved choline absorption compared to consumption of chemically produced choline bitartrate. This information is of relevance for the food industry, instead of adding choline-salts, adding choline from egg yolk phospholipids can improve choline uptake and positively impact health.

## 1. Introduction

Choline, a water-soluble vitamin-like essential nutrient, can affect diseases such as liver disease, atherosclerosis and neurological disorders [1]. Although the liver can produce small amounts of choline, choline needs to be obtained through the diet to avoid deficiencies [2]. The functions of choline are widespread; choline is mostly present as phosphatidylcholine, which is a phospholipid with a choline head group. Due to their amphiphilic properties, phospholipids form the lipid bilayer of all cell membranes, and are important for cell integrity. In addition to this, choline’s main metabolite, betaine, is an important source of methyl groups, essential in the one-carbon metabolism, which controls homocysteine concentrations [3]. Next, choline is a precursor for the neurotransmitter acetylcholine [4]. Though dietary sources of choline include eggs, beef, fish, pork, liver, soybean, and wheat germ [5]; choline intakes for children, adults, and pregnant women are often far below the adequate intake levels [1].

Especially during pregnancy and lactation choline consumption is very important. During these periods the demand for choline is high; plasma choline concentrations in pregnant women have shown to be 45% higher than in non-pregnant women [6], new-born infants have three times higher plasma choline concentrations than their mothers [6,7], and large amounts of choline are present in human milk [8,9,10]. In addition, Caudill et al., showed that maternal choline supplementation during pregnancy improved infant’s cognitive function [11]. Additionally, others concluded that choline consumption by pregnant women beneficially affects infants’ brain development [2,12,13,14], as well as neural tube development [2,13,14]. This shows that choline is essential for infant’s development. For that reason, in the US [15] and EU [16], infant formula must contain choline. To meet these recommendations, infant formulas derived from non-animal sources need added choline, since the raw materials contain no, or not enough, choline. Next, choline beneficially affects health and is, therefore, sold as a supplement, but is also added to functional foods. Nowadays, infant formula, supplement and functional food producers mostly add chemically-produced choline salts to their products, however, there is limited evidence that choline present in its natural form may be more efficiently absorbed [17] and, hence, improve its health impact.

In 1978, Hirsch et al. demonstrated that choline chloride caused a prompt increase in plasma choline concentrations (two-fold) after only 30 min of oral intake. Whereas consumption of natural choline bound to phospholipids showed a stronger increase in plasma choline, but a delayed kinetics compared with choline chloride [17]. However, this study has, to our best knowledge, never been reproduced and only choline responses were measured. Choline is rapidly absorbed [18], integrated into phospholipids and/or converted into betaine and dimethylglycine [19]. For bioavailability studies, measuring only free choline concentrations will not provide a good reflection of its absorption. Consequently, new studies are needed to compare the uptake of choline after natural phospholipid bound choline consumption compared with choline-salt intake. Such new evidence must also include responses of the choline metabolites, betaine and dimethylglycine in plasma. 

In addition to choline, another essential nutrient throughout lifespan is docosahexaenoic acid (DHA). DHA affects cognition, cardio vascular function, vision and inflammation throughout life [20]. Furthermore, in Europe it is mandatory to add DHA to infant formula, since the endogenous synthesis in infants is insufficient to maintain adequate tissue levels [16,21]. Most supplement, functional food and infant formula producers add fish or algae oil to their products, in which the DHA is bound to triacylglycerol (TAG). However, several studies point towards an improved DHA absorption after phospholipid-bound DHA compared with TAG-bound DHA consumption [22,23,24,25,26,27]. 

Egg yolks contain high amounts of natural choline, approximately 115 mg per serving [2], in the form of phosphatidylcholine, but they can also contain DHA bound to phospholipids. By feeding hens flaxseed, the egg yolk will be enriched with polyunsaturated fatty acids, especially DHA [28]. Most DHA will be bound to the phosphatidylcholine in the yolk, which makes these eggs an important natural source of phospholipid bound DHA that can be used for infant formula, supplements and functional foods. Adding egg yolk phospholipids has, therefore, the potential to improve both choline and DHA bioavailability.

The aim of this study was therefore to evaluate choline bioavailability in healthy adults after the consumption of an egg yolk phospholipid drink, containing natural phospholipid bound choline in the form of phosphatidylcholine, and compare this with a control drink containing chemically produced choline bitartrate. Additionally, DHA bioavailability was measured after consumption of the egg yolk phospholipid drink, containing phospholipid-bound DHA and compared it with a control drink containing TAG-bound DHA from algae oil. 

## 2. Subjects and Methods

### 2.1. Ethics Statement 

This trial has been reviewed and approved by the Medical Ethics Committee of Wageningen. Furthermore, the study was conducted according to the principles of the Declaration of Helsinki, in accordance with the Medical Research Involving Human Subjects Act (WMO) and registered at Clinical Trials.gov (identifier NCT 67054.081.18). All subjects gave their written informed consent before entering the study.

### 2.2. Subjects 

Eighteen apparently healthy adults (*n* = 7 men and 11 women) with an age range between 30–70 years (mean ± SD = 62.3 ± 7.2 years) and a BMI between 18.5 and 24.9 kg/m^2^ were recruited from the surroundings of Wageningen. All subjects were non-smoking, did not have any metabolic, gastrointestinal, inflammatory or chronic disease, or a history of gastro-intestinal surgery or (serious) gastro-intestinal complaints. Subjects did not use choline or fish oil supplements, or any medication or food supplements known to interfere with the study results, such as laxatives, stomach protectors and drugs that can affect intestinal motility. 

### 2.3. Study Design

The study was a randomized, cross-over, double blind, controlled trial. Study participants visited the research facility on two occasions under fasting conditions with a washout period of one week between the two study visits. On the day prior the study day, all study participants received a standardized dinner and were instructed not to eat or drink anything except water after 08.00 pm. The participants were also instructed not to drink alcohol, perform heavy exercise and to avoid choline rich products (e.g., animal products like eggs, dairy, fish or meat).

**Figure 1:**
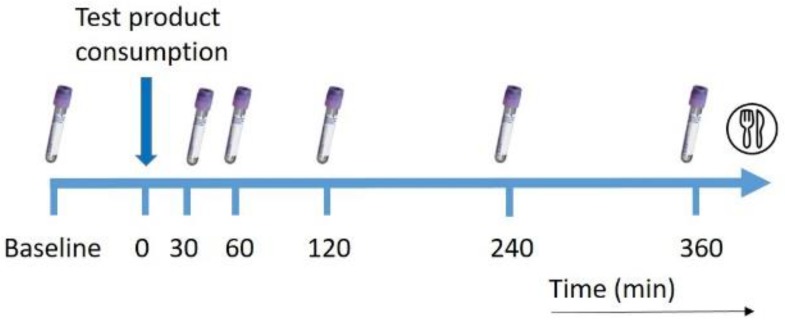
Overview of blood collection time points during a test day.

During each visit, study subjects received either a drink with natural choline from egg yolk phospholipids (ELIP, AAK), which included phosphatidylcholine and DHA bound to this phosphatidylcholine, or a control shake with choline added as a salt (bitartrate) and DHA added as algae oil (TAG-bound). The two test drinks were administered in randomized order by block randomization and blood samples were collected via a catheter before the drink and 30, 60, 120, 240 and 360 min after consumption of the drink (Figure 1). Plasma samples were stored at −80 °C until further analysis. 

### 2.4. Intervention Drinks

The egg yolk phospholipid drink contained 37 g freeze-dried egg phospholipids (equivalent of 3 g choline, ELIP, AAK, the Netherlands, Table 1) and 4 g high oleic sunflower oil (AAK, the Netherlands), while the control drink contained 9.3 g choline bitartrate (equivalent of 3 g choline, Bulkpowders, the Netherlands) and 41 g of oil blend (37 mL high oleic sunflower oil, 1 g DHA and 2 g arachidonic acid (AAK, the Netherlands). Arachidonic acid was added to the control drink to control for the amount of arachidonic acid bound to the phospholipids in the egg yolk phospholipid drink.

Both drinks contained 230 g commercially available mango juice (Albert Heijn, the Netherlands), 20 g whey protein isolate (Myprotein, the Netherlands) and 40 g maltodextrin (Myprotein, the Netherlands). Choline and DHA were added to the control drink to match the content already present in the egg yolk phospholipid drink. However, DHA in the control drink was 1.3 times higher compared to the egg yolk phospholipid drink. The volume of both drinks was approximately 330 mL. Macronutrient composition of the test and control drinks is listed in Table 2.

### 2.5. Quantification of Choline, Betaine and Dimethylglycine in Plasma

The assay used d_9_-betaine chloride, d_9_-choline chloride, and d_6_-dimethylglycine HCl as internal standards. EDTA plasma samples (100 µL) were diluted using 300 µL internal standard mix dissolved in acetonitrile. After precipitation of the proteins, the samples were centrifuged for 5 min at 10,000× g at room temperature and the supernatant was transferred to glass vials. Sealed vials were immediately measured using an Acquity Ultra Performance LC system coupled to a MicroMass Quattro Premier XE tandem quadrupole mass spectrometer (Waters Corporation, Milford, MA, USA). The analytes were separated on an Acquity UPLC BEH HILIC column (100 mm × 2.1 mm (i.d.); 1.7 µm particle size) with an Acquity HILIC VanGuard pre-column (5 mm × 2.1 mm (i.d.); 1.7 µm particle size) and a 0.2 µm in-line filter (Waters Corporation). The column temperature was 30 °C and the flow rate was 0.6 mL/min. The separation was performed using ammonium formate (solvent A) and acetonitrile (solvent B) as described in detail [29]. The interassay CVs were ≤7% for betaine and choline, and 9.0% for dimethylglycine. The measurements of choline, betaine and dimethylglycine were performed at the Central Laboratory of the University Hospital of the Saarland, Germany.

### 2.6. DHA in TG and Phospholipid Fraction

DHA concentrations were quantified in the triglyceride and phospholipid fractions of EDTA plasma using a modified version of a previously described protocol [30]. In short, 650 µL EDTA plasma was extracted using hexane in the presence of C19:0 TAG and C19:0 PL internal standards, and lipids were purified by solid phase extraction using silica columns. Subsequently, fatty acid methyl esters are formed, which were analysed using gas chromatography coupled to flame ionization detection (GC–FID) as described before. Concentrations were calculated via single-point calibration, using the peak area from the C19 internal standards as calibrator. The inter-assay CVs were 22% for the triglyceride (spike 8%) and 14% for the phospholipid fractions (spike 13%).

### 2.7. Statistical Analysis 

Statistical analysis was performed using linear mixed models for repeated measures (IBM SPSS Statistics 23.0; IBM, Armonk, NY, USA). Effect of the intervention was determined using treatment (test drink or control drink) and time (in minutes) as fixed effects, and treatment × time interaction. If this interaction term was not significant, it was omitted from the model. All statistical analysis were performed on delta values in order to correct for baseline differences. Incremental area under the curve (iAUC) values were calculated for each individual by using the trapezoidal method [31]. Differences in iAUC between interventions were analysed by a paired *t*-test. A *p*-value < 0.05 was considered to be significant.

## 3. Results

### 3.1. Subject Characteristics

All 18 subjects completed the study. The baseline characteristics of these 18 participants are summarized in Table 3. Evaluation of adverse events (AEs) demonstrated that some individuals experienced a change of fecal consistency). We recorded seven cases; one participant during both intervention days, four participants after consumption of the egg yolk phospholipid drink and one participant after consumption of the control drink with choline-bitartrate.

### 3.2. Choline, Betaine and Dimethylglycine Responses 

**Figure 2:**
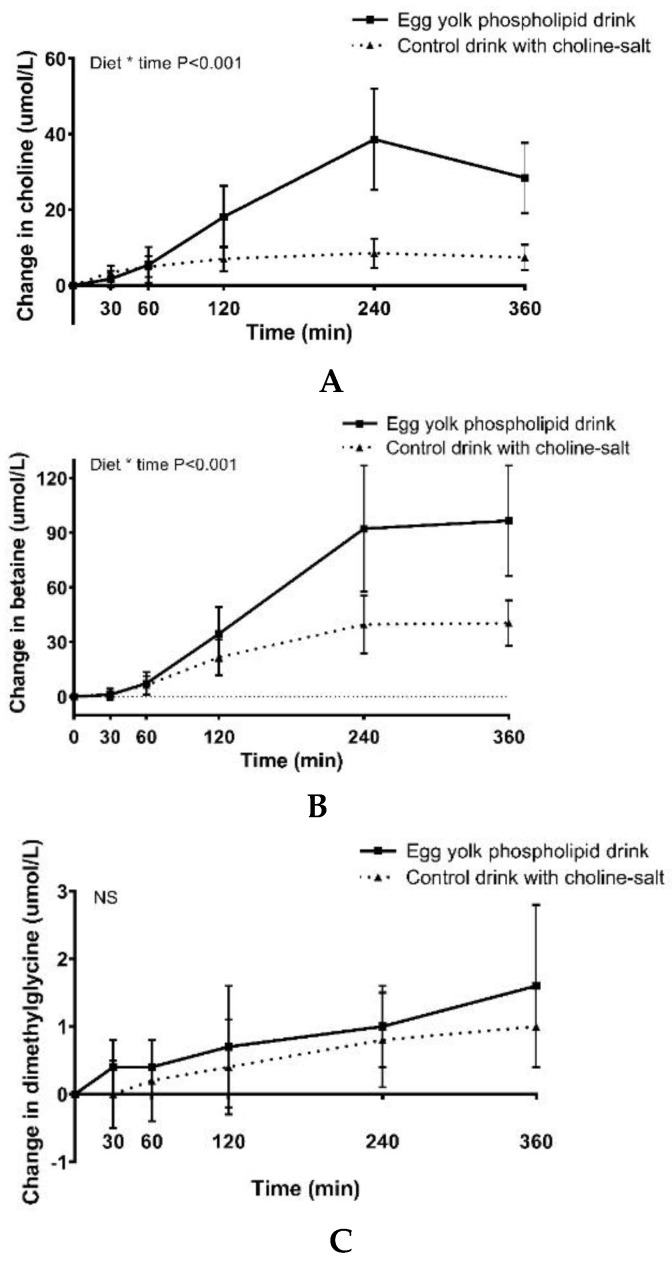
Mean changes from baseline in plasma (**A**) choline, (**B**) betaine and (**C**) dimethylglycine concentrations after egg yolk phospholipid and choline bitartrate consumption. Data are reported as mean changes ± SD, *n* = 18. Linear mixed model procedures were conducted to determine difference between the responses.

Plasma choline, betaine and dimethylglycine concentrations after consumption of both treatments showed different response curves (Figure 2A–C, Table S1). Baseline choline concentrations did not differ between the two test days (*p =* 0.36). For changes in postprandial choline values, the treatment × time interaction was significant (*p <* 0.01). The concentrations of choline increased more pronounced after consumption of the egg yolk phospholipid drink compared to the control drink with choline bitartrate. When expressed as iAUC over the 6-hour time period, the egg yolk phospholipid drink showed a 4 times higher response compared to the control drink with choline bitartrate (*p* < 0.01, Table 4). Although choline plasma concentrations did not yet return to baseline values after six hours, concentrations started to decrease.

**Figure 3:**
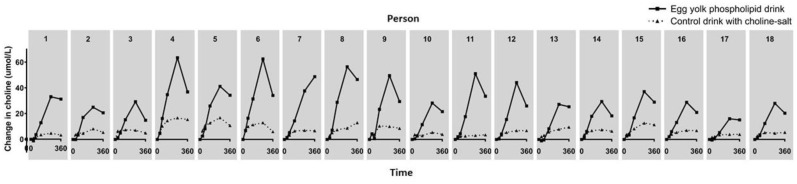
Individual choline responses after egg yolk phospholipid or choline bitartrate consumption. Numbers 1–18 represent individual study participants.

Individual response curves clearly demonstrated that these responses were very consistent among individual study participants (Figure 3). Response curves of the individuals that experienced a change of fecal consistency showed no deviating pattern, suggesting that this had no effect on choline uptake.

Upon uptake, choline is rapidly converted into its metabolites betaine and dimethylglycine. Plasma concentration of betaine showed similar responses as choline. Again, the treatment*time interaction was significant, with a more pronounced increase after consumption of the egg yolk phospholipid drink compared to the control drink with choline bitartrate (*p <* 0.01). This was also confirmed when expressed as iAUC over the six-hour time period (Table 3). Additionally, dimethylglycine levels were higher after consumption of the egg yolk phospholipid drink compared to the control drink with choline bitartrate, but that difference was not as profound as with choline and betaine (Figure 2C, Table 3).

### 3.3. Responses in Plasma DHA in TG and Phospholipid Fraction 

Baseline and postprandial concentrations of plasma DHA in the TAG and phospholipid fraction are shown in Supplemental Table 1. DHA concentrations in the phospholipid fraction did not show a postprandial response after consumption of both drinks. Plasma DHA concentrations in the TAG fraction increased more pronounced after consumption of the control drink with TAG bound DHA compared to the egg yolk phospholipid drink with phospholipid bound DHA. When expressed as iAUC over the 6-hour time period, the control drink showed a 1.5 times higher response compared to the egg yolk phospholipid drink (*p* = 0.01, Table 3). However, the administered dose of DHA differed between the two test drinks, the DHA content in the control drink was 1.3 times higher than the dose in the egg yolk phospholipid drink. Changes in plasma concentrations of DHA in the TAG fraction were not different between the test drinks when corrected for this dose difference. 

## 4. Discussion 

This randomized, double blind, controlled bioavailability study showed that the plasma concentrations of choline and choline’s main metabolites, betaine and dimethylglycine, were significantly higher after intake of an egg yolk phospholipid drink with natural choline compared with a drink with similar amounts of chemically produced choline bitartrate. Changes in plasma DHA concentrations in the TAG and phospholipid fraction, were not different between the egg yolk phospholipid drink with phospholipid bound DHA and the control drink with TAG bound DHA when corrected for the difference in administered dose.

Our results clearly demonstrate that choline is better absorbed when it is consumed in the natural form; choline absorption was 4 times higher comparing egg yolk phospholipid consumption with choline bitartrate intake, as determined by the iAUC. This difference in absorption was highly significant and very consistent among participants. Choline’s main metabolites, betaine and dimethylglycine, showed similar outcomes. Plasma concentrations of both betaine and dimethylglycine were significantly increased after egg yolk phospholipid consumption compared with choline bitartrate intake. 

These outcomes are in line with the results of Hirsch et al. [17], who also found that the choline uptake over time is more efficient when consumed as phospholipid than as choline chloride. In both studies the response of the choline salt was higher in the first half hour, while later in time the phospholipid-bound choline showed an improved absorption. Although there was a difference, Hirsch and colleagues found that the plasma choline concentrations were still increasing 12 hours postprandially, while concentrations in our study already decreased four hours after consumption of the drinks. This is probably due to the difference in meal composition between the studies. In the study of Hirsch et al., participants received liquid together with solid foods [17], while our study participants consumed solely a liquid drink. Liquid foods have a faster gastric emptying rate [32], hence, a faster absorption rate. Furthermore, in accordance with our results, a more recent study by Lemos et al. demonstrated that fasting choline concentrations increased 20% more after four-week egg intake compared to choline bitartrate supplementation (both 400 mg choline per day). Contrary, in their study, plasma choline concentrations did not increase after choline bitartrate consumption, and plasma betaine concentrations did not rise at all, while in our study both choline and betaine concentrations increased after the two treatments. This difference can probably be explained by the lower choline dose used in the study of Lemos and colleagues [33]. 

Our results, together with the studies of Hirsch et al., and Lemos et al., clearly point towards an improved choline bioavailability after natural choline consumption from phospholipids compared with choline-salt intake. It seems that natural phosphatidylcholine, which is especially high in egg yolk, improves choline absorption. We can only speculate about the underlying mechanism of this improved bioavailability. Perhaps the difference in absorption can be explained by the difference in choline carriers, e.g., phospholipids and salts, since both have different digestion and absorption routes. Most dietary phospholipids are hydrolysed in the small intestine, resulting in a lysophospholipid and a fatty acid part [34,35]. Via passive diffusion or via the formation of micelles, the lysophospholipids are directly absorbed by the intestinal epithelium [34,35]. When choline is bound to a salt, the choline is transported by a saturable carrier system via passive diffusion in a concentration dependent manner [36]. Since the choline carrier is saturable, choline intake as a salt, may lead to a less efficient choline absorption than after the consumption of phosphatidylcholine, especially when consumed in high concentrations. When choline is not absorbed in the small intestine, it will reach the colon where it will be available for the microbiota and converted to trimethylamine. Higher levels of trimethylamine are associated with an increased risk of cardio vascular diseases [37]. Theoretically, faster absorption rates may result in less choline available for the microbiota, hence preventing the formation of trimethylamine. 

While levels of plasma choline started to decline after 4 hours, those of betaine seemed to reach a steady state and the response of dimethylglycine seems not to reach its maximum by the time the observation time window ended (six hours). Our results show a time-shift between the responses of the three metabolic markers. These results mean that choline from egg yolk has reached the circulation and the liver, where it can be used in several metabolic pathways. Next free choline from egg yolk can undergo mitochondrial oxidation to betaine by choline dehydrogenase and betaine aldehyde dehydrogenase. Through this oxidation to betaine, choline from egg yolk phospholipids can provide methyl groups coming from the betaine to dimethylglycine conversion via betaine homocysteine methyl transferase. Through this reaction, homocysteine is converted to methionine. Since dimethylglycine concentrations were also significantly higher after egg yolk phospholipid consumption than after choline bitartrate intake, this could mean that choline from egg yolk phospholipids is a better source of methyl groups and its effect lasted for a longer time than choline bitartrate. Repeated consumption of egg yolk phospholipid choline could therefore improve liver function in patients with fatty liver [38] or lowering plasma homocysteine, hence impacting cardiovascular disease risk [39]. In addition, the enhanced methyl group production from egg yolk phospholipids can provide *S*-adenosylmethionine that may feed the *S*-adenosylmethionine-dependent phosphatidylethanolamine *N*-methyltransferase (PEMT) that synthesize phosphatidylcholine from phosphatidylethanolamine. Next, the egg yolk phospholipids also contained some phosphatidylethanolamine, which can also be converted to phosphatidylcholine when *S*-adenosylmethionine is made available. The greater increase in methyl groups after egg yolk phospholipid consumption together with the phosphatidylcholine made from phosphatidylethanolamine can lead to higher conversion rates of choline to betaine. This will subsequently increase betaine concentrations in the liver and, finally, also in the circulation, which might explain the greater change in betaine than in choline as a function of time.

In this study plasma DHA concentrations in the phospholipid and TAG fraction were measured. We expected to find an increase in DHA concentrations in the phospholipid fraction after consumption of the egg yolk phospholipid drink, since in this drink DHA was bound to phospholipids. However, no postprandial increase of DHA in the phospholipid fraction appeared. The fatty acid composition in the phospholipid fraction reflects dietary intake of hours to weeks [40,41], which may explain why we did not find changes in this fraction only a few hours after a single intake. Next, we expected an increase in DHA in the TAG fraction after intake of the control drink, since in this drink DHA was bound to TAG. The DHA concentrations in the TAG fraction did increase after consumption of the drinks, with a greater increase after consumption of the control drink compared with the egg yolk phospholipid drink. This can be explained by the higher DHA dose in the control drink than in the egg yolk phospholipid drink. We found that the difference in iAUC largely matched with the difference in administrated dose. We, therefore, conclude that there was no difference in DHA absorption between the two drinks. 

Still, several studies point towards an improved DHA absorption after phospholipid bound DHA compared with TAG bound DHA consumption. These studies investigated the effects after prolonged intake of at least 4 weeks [22,23,24,25,26] or, if tested in a postprandial study, used a very high dose of DHA (1810 mg) [27]. Most likely, sampling time in our study was too short and the single dose of DHA was too low to find this expected difference in DHA absorption between DHA bound to a phospholipid or TAG as has been shown in previous studies.

For infants, phospholipid bound DHA seems to be of great importance. In human milk, phospholipids represent only 2% of the total lipids. However, 13% of the DHA in human milk is bound to this small phospholipid fraction [42]. Therefore, phospholipid bound DHA seems to be important for infant development. Recent evidence also indicates that the synergy of choline and DHA, when included in a single product, is of importance for infant development. Cheatham and colleagues demonstrated in human infants that high concentrations of both DHA and choline in milk improved infant’s cognition compared with milk only high in DHA or choline [43]. Additionally, in preterm infants, supplementation with DHA alone increased DHA in the phospholipid fraction with 35%, while in combination with choline it increased up to 63% [44]. A recent study showed similar effects in adult mice, supplementation of choline, (with ribose, pyrophosphate and cytosine), together with DHA increased the amount of DHA containing phosphatidylcholine in the brain and improved learning and memory ability compared with DHA or choline supplementation alone [45]. The mechanism behind such a DHA-choline synergy can be explained by the structural properties of HDL particles. HDL particles are high in phosphatidylcholine and transport DHA to the brain. Higher consumption of phosphatidylcholine augments the number of HDL particles, but also the amount of DHA incorporated within the HDL particles [43,44]. Furthermore, DHA can only cross the blood brain barrier when bound to a phospholipid [46]. Thus, after the consumption of DHA together with phosphatidylcholine, more DHA can be transported within HDL particles and subsequently can enter the brain. Consumption of egg yolk phospholipids, containing phosphatidylcholine-bound DHA, may, therefore, be beneficial for infants. 

Considering the application of choline in infant formula, a limitation of the current study is that it was only performed in healthy adults, not in infants. However, performing this kind of clinical trials in infants is ethically not feasible. It is, however, highly unlikely that choline absorption differs between infants and adults [18,36]. Therefore, absolute concentrations will not be comparable, but the difference in uptake between the different drinks can most likely be extrapolated to infants. Although not uncommon in bioavailability studies, another aspect to consider when interpreting our results is that the dose of choline provided is higher than during normal daily consumption. The European Food and Safety Authority (EFSA) recommends an adequate intake of 115 mg/day for infants, 140–400 mg/day for children, 400 mg/day for adults, 450 mg/day for pregnant women and 550 mg/day for lactating women [47]. The tolerable upper intake level for choline for adults is 3.5 g/day [47]. In this study we provided a single dose of 3 g of choline, which is a relatively high dose, but lower than the Tolerable Upper Intake Level. We cannot rule out that the current used high dose may have overloaded the saturable choline carrier system. Theoretically, it may, therefore, be possible that different results can be expected when administering a lower dose. However, we think that this is unlikely since Lemos and colleagues [33] demonstrated comparable results with a lower dose of 400 mg/d.

Another important point of attention is that some individuals experienced a change of faecal consistency during the trial. Although the number of reported cases of diarrhoea was higher in the intervention group, the diarrhoea did not affect the study results. 

Strong points of the study were the sample size, study design and low carry over effect. A sample size of 18 subjects in cross over design and with low variation in choline response between participants, gave a very clear outcome. Our sample size was large enough to detect differences in our primary outcome, e.g., choline responses. Unfortunately, the study was not optimally designed to detect differences in DHA concentrations between the drinks, since the duration was most likely too short, and the dose was too low and not similar between the two test drinks. 

## 5. Conclusions

The consumption of natural choline from egg yolk phospholipids improved choline absorption compared to consumption of chemically produced choline bitartrate. Apparently, the matrix in which choline is consumed is important for its uptake. This information can be particularly relevant for the development of infant formula, supplements and functional foods. Instead of adding choline as a salt, adding choline from egg yolk phospholipids can improve choline uptake and, thereby, has a positive impact on health.

## Figures and Tables

**Table 1:** Phospholipid content of the egg phospholipids (ELIP, AAK).

	ELIP (wt%)
Phospholipids	75
Phosphatidylcholine	59
Phosphatidylinositol	1
Sphingomyelin	2
Phosphatidylethanolamine	11
Phosphatidylserine	0

**Table 2:** Macronutrient composition of the two test drinks.

	Egg Yolk Phospholipid Drink	Control Drink Choline-Bitartrate
Energy (kcal)	712	714
Fat (g)	41	41
Fat (E%)	52	52
Carbohydrates (g)	67	67
Carbohydrates (E%)	38	38
Protein (g)	19	19
Protein (E%)	10	10
Choline (g)	3.0	3.0
DHA (mg)	595	787

DHA; docosahexaenoic acid.

**Table 3:** Baseline characteristics of the subjects.

	*(n = 18)*
Male/female, *n* (%)	7/11
Age (years), Mean ± SD	62.3 ± 7.2
BMI (kg/m^2^), Mean ± SD	22.8 ± 2.0

**Table 4:** iAUC and P values for choline, betaine, dymethylglycine, DHA-PL, and DHA-TG for the total 6 h postprandial responses of the egg yolk phospholipids drink compared with the control drink containing choline bitartrate.

	Intervention		
	Egg Yolk Phospholipid Drink	Control Drink with Choline Bitartrate	*p* Value
Choline (µmol/L/min)	8020 ± 2967	2424 ± 1112	<0.01
Betaine (µmol/L/min)	20351 ± 7350	9455 ± 3603	<0.01
Dimethylglycine (µmol/L/min)	309 ± 196	215 ± 161	0.03
DHA-PL (mg/ml/min) *	5 ± 6	4 ± 5	0.63
DHA-TG (mg/ml/min) *	4 ± 1	6 ± 2	0.01

Mean ± SD, *n* = 18. iAUC; incremental area under the curve, DHA-PL; phospholipid bound docosahexaenoic acid, DHA-TG; triglyceride bound docosahexaenoic acid. * Administered DHA concentrations were higher in the control drink.

## Supplementary Materials

The following are available online at https://www.mdpi.com/2072-6643/11/11/2758/s1, Table S1: Baseline values and changes in choline, betaine, dimethylglycine, phospholipid-bound DHA, and TAG-bound DHA after consumption of the egg yolk phospholipid drink compared with the control drink with choline bitartrate.

## Abbreviations

DHA	docosahexaenoic acids
TAG	triacylglycerol
