# Supplementary material for: Natural Choline from Egg Yolk Phospholipids Is More Efficiently Absorbed Compared with Choline Bitartrate; Outcomes of A Randomized Trial in Healthy Adults
Source: Nutrients. 2019 Nov 13;11(11):2758. doi: 10.3390/nu11112758 (PMC6893749; doi:10.3390/nu11112758)
Supplement: Supplementary file 1 [file nutrients-11-02758-s001.pdf]

**Supplemental Table 1:** Baseline values and changes in choline, betaine, dimethylglycine, phospholipid-bound DHA, and TAG-bound DHA after consumption of the egg yolk phospholipid drink compared with the control drink with choline bitartrate

| | | Time (min) | | | | | | P-Value | | |
|---|---|---|---|---|---|---|---|---|---|---|
| | | Baseline | 30 | 60 | 120 | 240 | 360 | Time | Intervention | Interaction |
| Choline (μmol/L) | Egg yolk phospholipid drink | 10.5 ± 3.9 | 12.2 ± 5.0 | 15.9 ± 6.7 | 28.5 ± 10.8 | 49.0 ± 15.9 | 38.9 ± 11.9 | $p < 0.01$ | $p < 0.01$ | $p < 0.01$ |
| | Control drink with choline-salts | 10.1 ± 4.1 | 13.6 ± 5.3 | 15.0 ± 6.3 | 17.1 ± 6.9 | 18.6 ± 7.6 | 17.5 ± 6.8 | | | |
| Betaine (μmol/L) | Egg yolk phospholipid drink | 40.0 ± 16.8 | 41.1 ± 16.9 | 47.4 ± 18.0 | 74.5 ± 25.1 | 132.3 ± 44.3 | 136.6 ± 39.8 | $p < 0.01$ | $p < 0.01$ | $p < 0.01$ |
| | Control drink with choline-salts | 38.7 ± 11.1 | 40.1 ± 11.7 | 45.0 ± 13.1 | 60.3 ± 17.5 | 78.3 ± 22.3 | 79.1 ± 19.2 | | | |
| Dimethylglycine (μmol/L) | Egg yolk phospholipid drink | 3.6 ± 0.8 | 3.9 ± 1.0 | 4.0 ± 1.0 | 4.3 ± 1.0 | 4.5 ± 1.0 | 5.2 ± 1.0 | $p < 0.01$ | NS | NS |
| | Control drink with choline-salts | 3.9 ±0 .5 | 3.9 ± 0.7 | 4.0 ± 0.7 | 4.3 ± 0.8 | 4.6 ± 0.7 | 4.9 ± 0.7 | | | |
| DHA -PL (mg/ml)* | Egg yolk phospholipid drink | 0.17 ± 0.11 | 0.16 ± 0.06 | 0.16 ± 0.05 | 0.16 ± 0.06 | 0.16 ± 0.06 | 0.17 ± 0.06 | NS | NS | NS |
| | Control drink with choline-salts | 0.16 ± 0.06 | 0.17 ± 0.07 | 0.16 ± 0.07 | 0.16 ± 0.06 | 0.16 ± 0.06 | 0.18 ± 0.09 | | | |
| DHA -TAG (mg/ml)* | Egg yolk phospholipid drink | 0.01 ± 0.01 | 0.01 ± 0.01 | 0.01 ± 0.01 | 0.02 ± 0.01 | 0.03 ± 0.01 | 0.03 ± 0.01 | $p < 0.01$ | $p < 0.01$ | $p < 0.01$ |
| | Control drink with choline-salts | 0.01 ± 0.01 | 0.01 ± 0.01 | 0.02 ± 0.01 | 0.03 ± 0.01 | 0.03 ± 0.02 | 0.04 ± 0.02 | | | |

Mean ± SD. *n*=18.DHA-PL; phospholipid bound docosahexaenoic acid. DHA-TG; triglyceride bound docosahexaenoic acid. NS not significant. *Administered DHA concentrations were higher in the control drink.
